# Identification of LINC00654-NINL Regulatory Axis in Diffuse Large B-Cell Lymphoma *In Silico* Analysis

**DOI:** 10.3389/fonc.2022.883301

**Published:** 2022-05-26

**Authors:** Yinchu Chen, Chen Li, Nana Wang, Zhenghao Wu, Jin Zhang, Jiawei Yan, Yuanfeng Wei, Qunlong Peng, Jing Qi

**Affiliations:** ^1^Department of Hematology, The First Affiliated Hospital of Wannan Medical College, WuHu, China; ^2^Department of Biology, Chemistry, Pharmacy, Free University of Berlin, Berlin, Germany; ^3^Department of Clinical and Surgery, Moscow State First Medical University, Moscow, Russia; ^4^Department of Surgery, I.M. Sechenov First Moscow State Medical University, Moscow, Russia; ^5^College of Pharmacy, Xiangnan University, Chenzhou, China

**Keywords:** DLBCL, NINL, random forest, LINC00654, mechnism

## Abstract

**Background:**

The long non-coding RNA (lncRNA)-mRNA regulation network plays an important role in the development of diffuse large B-cell lymphoma (DLBCL). This study uses bioinformatics to find an innovative regulation axis in DLBCL that will provide a positive reference for defining the mechanism of disease progression.

**Methods:**

Batch Cox regression was used to screen prognosis-related lncRNAs, and a random forest model was used to identify hub lncRNA. The clinical value of the lncRNA was evaluated and Spearman correlation analysis was used to predict the candidate target genes. Gene Oncology (GO) and Kyoto Encyclopedia of Genes and Genomes (KEGG) pathway enrichment were used to define the biological function of the lncRNA. A batch Cox regression model, expression validation, and Spearman correlation analysis were used to select the best downstream target genes. The expression and prognostic value validation of this gene was conducted using public data. Gene Set Enrichment Analysis (GSEA) was performed to explore potential mechanisms for this gene in DLBCL.

**Results:**

LINC00654 was identified as the hub lncRNA and 1443 mRNAs were selected as downstream target genes of the lncRNA. The target genes were enriched in the regulation of GTPase and Notch signaling pathways. After validation, the ninein-like (NINL) gene was selected as the potential target of LINC00654 and the LINC00654-NINL axis was constructed. Patients with better responses to therapy were shown to have high NINL gene expression (p-value = 0.036). NINL also had high expression in the DB cell line and low expression in the OCILY3 cell line. Survival analysis showed that high NINL expression was a risk factor for overall survival (OS) and disease-specific survival (DSS) within older patients and those with advanced-stage cancer. GSEA results showed that NINL may be involved in neutrophil-mediated immunity and NF-κB signaling.

**Conclusion:**

This study identified a novel LncRNA00654-NINL regulatory axis in DLBCL, which could provide a favorable reference for exploring the possible mechanisms of disease progression.

## Introduction

Diffuse large B-cell lymphoma (DLBCL) is an aggressive lymphoma that is responsible for 30%–35% of non-Hodgkin lymphoma (NHL) in adults (1). Although treatment strategies should be stratified by risk group and physical status, the most common up-front treatment is a combination of immunotherapy and chemotherapy containing R-CHOP (rituximab, cyclophosphamide, doxorubicin, vincristine, and prednisone), which cures about 60% of patients. Unfortunately, the remaining third of patients fail to respond to treatment or relapse after treatment completion (2, 3). Thus, it is critical to explore the specific mechanism of disease progression and find potential therapeutic targets.

LncRNAs are a type of RNA that are defined as gene transcripts that are not translated into protein. Instead, they regulate target protein-coding genes at multiple levels and influence cell proliferation, survival, migration, and genomic stability (4, 5). While the intrinsic function of most lncRNAs remains to be explored, some mechanisms of action have been defined (6). LncRNAs can recruit the chromatin-remodeling complex to a specific DNA region to mediate epigenetic modification, and can also modify transcription factor activity to regulate the transcription process. During the post-transcription process, they regulate mRNA through capping, splicing, editing, and degradation. During this complicated regulatory network, various events can disrupt cellular homeostasis and lead to disease and tumorigenesis. Studies of the genetic background of cancer indicate that the deregulation of the lncRNA-mRNA network is strongly associated with tumorigenesis and tumor progression in a variety of cancer types (7, 8), and some lncRNA-mRNA axes are shown to play an important role in the occurrence and development of DLBCL. For example, the lncNBAT1-APOBEC3A network mediates HBX-induced chemoresistance and lncRNA TUG1 plays an oncogenic role by inhibiting MET ubiquitination (9). Some integrated lncRNA-mRNA signatures are also used for DLBCL prognosis (10). These findings support the use of the lncRNA-mRNA regulatory network as a potential predictive biomarker for DLBCL.

The current study identified a novel prognostic lncRNA, LINC00654, using public datasets and revealed its potential biological function in DLBCL through enrichment analysis of its co-expressed downstream genes. These downstream target genes were also predicted through batch survival analysis and expression validation, and the LINC00654-NINL mRNA regulatory axis was identified. LINC00654 is located on chromosome 20p12.3 and is reported as a risk lncRNA in several cancers (8, 11, 12). However, the role of LINC00654 and its regulatory network in the progression of DLBCL remains unknown. Ninein-like protein (Nlp), encoded by the NINL gene, was identified as a key regulator of centrosome maturation, spindle formation, and chromosome separation (13, 14). Previous studies have shown that the centrosome plays a critical role in regulating mitosis events through all stages of the cell cycle. Aberrations may cause cell cycle progression, cell transformation, and tumorigenesis (15). Although upregulated NINL mRNA and protein expression have been observed in several types of human cancer (16–21), the potential function and prognostic role of NINL in DLBCL remains unknown. This study is the first to describe a potential role for the LINC00654-NINL mRNA regulatory axis in DLBCL, which may provide a valuable reference for exploring possible mechanisms of disease progression.

## Methods

### Public Data Acquisition and Preprocessing

RNA sequence and corresponding clinical data from 47 DLBCL patients were downloaded from The Cancer Genome Atlas (TCGA) database (https://portal.gdc.cancer.gov/). Patients were only included in the study if they had complete clinical follow-up data, including age, sex, histological type, and tumor stage. The GSE32018 and GSE18376 datasets were downloaded from the Gene Expression Omnibus (GEO) database for validation, and cell line data wereobtained from the Cancer Cell Line Encyclopedia (CCLE) database.

### Screening Prognosis-Related LncRNAs

Batch Cox regression was performed to screen for independent risk lncRNAs that affect patient prognosis. Patients with a survival time < 30 days were excluded. LncRNAs with a P-value <0.05 were considered candidate genes.

### Selecting the Hub LncRNA

After regression analysis, the top three lncRNAs were identified as candidate lncRNAs. TCGA and GTExPortal data were used to compare the expression of the three genes between tumor and normal samples. Significant Cox regression results were also included in the random forest model and the importance of this model was set to >0.3 to obtain the hub LncRNA. By combining expression validation and random forest results, the hub LncRNA was selected.

### Evaluating the Clinical Value and Selected Target Genes

Clinical factors play an important role in patient outcomes. This study compares the gene distribution among different patient groups by clinical factors to assess the potential clinical value of hub lncRNA. Spearman correlation analysis was used to identify potential target mRNAs of the hub lncRNA, using a correlation >0.4, and a P- value <0.05.

### Function Annotation and Pathway Enrichment Analysis

To predict the possible function of hub lncRNA in DLBCL, Gene Oncology analysis was performed using target genes that were resourced from Spearman correlation analysis results. These co-expression genes were also used for KEGG pathway enrichment analysis to define the pathways used by this gene.

### Prognosis-Related Target Genes of LncRNA

DSS was used to find more valuable endpoints of lncRNA. OS and PFI were used as critical references. Batch Cox regression was used to predict DSS, OS, and PFI. Significant DSS-related genes were selected and the ability of these genes to prognose OS and PFI was evaluated.

### Identification of Key Target Genes and Construction of the LncRNA-mRNA Axis

The expression of significant genes was compared between tumor and normal samples using TCGA and GTEx Portal data. Gene expression trends consistent with lncRNA between two groups along with high correlation coefficients were selected as the final candidate target.

### Expression Verification of the Candidate Target Gene

The GSE32018 dataset was used as a validation dataset to verify candidate target gene expression, and TCGA samples were used to conduct the age and stage subgroup analysis. The GSE18376 dataset was used to verify the response to therapy. Target gene expression in DLBCL cell lines was validated using the CCLE database.

### Gene Set Enrichment Analysis

Using the median expression of the target gene, samples were divided into high and low expression groups. Gene set enrichment analysis (GSEA) was then used to explore the biological processes (BPs), cellular components (CCs), and molecular functions (MFs) of the target gene in DLBCL. Pathways related to the target gene were also conducted using GSEA.

### Statistical Analysis

R software (Version 4.0.3) was used to perform the statistical analyses. Cox regression analysis was used to select the independent prognosis-related lncRNAs in DLBCL patients. A random forest model was used to select the lncRNA related to patient prognosis. Survival analysis was estimated using the log-rank and Wilcoxon tests, and the Kruskal–Wallis test was used to assess differences between stratified groups. Statistical significance was defined as a two-tailed p-value <0.05.

## Results

### Clinical Features of DLBCL in the TCGA Database

The gene-expression profiles of 48 DLBCL patients were obtained from the TCGA database. Patient clinical characteristics are summarized in **Table 1**. The GSE32018 dataset (22 DLBCL samples and seven normal lymph-node tissues) and the GSE18376 dataset (23 DLBCL samples) were used to verify expression and evaluate the response to therapy. Six DLBCL cell lines from the CCLE database were also used to verify target gene expression in cell lines.

**Table 1:** Baseline characteristics of DLBCL patients from the TCGA database.

Characteristic	Levels	Overall	
		N=48	%
**Age (%)**	<60	27	56.2%
	≥60	21	43.8%
**Gender (%)**	Female	26	54.2%
	Male	22	45.8%
**Race (%)**	Asian	18	37.5%
	White	29	60.4%
	Other	1	2.1%
**Weight (%)**	<=70	27	56.2%
	>70	21	43.8%
**Height (%)**	<=165	26	54.2%
	>165	22	45.8%
**BMI (%)**	<=25	25	52.1%
	>25	23	47.9%
**Clinical stage (%)**	Stage I	8	16.7%
	Stage II	17	35.4%
	Stage III	5	10.4%
	Stage IV	12	25%
	Unknown	6	12.5%
**Therapy outcome (%)**	PD^a^	5	10.4%
	SD^b^	3	6.25%
	PR^c^	3	6.25%
	CR^d^	35	72.9
	Unknown	2	4.2%

^a^PD, progressive disease; ^b^SD, stable disease^c^PR, partial response; ^d^CR, complete response.

### Survival-Related LncRNAs and Expression Validation

**Figure 1:**
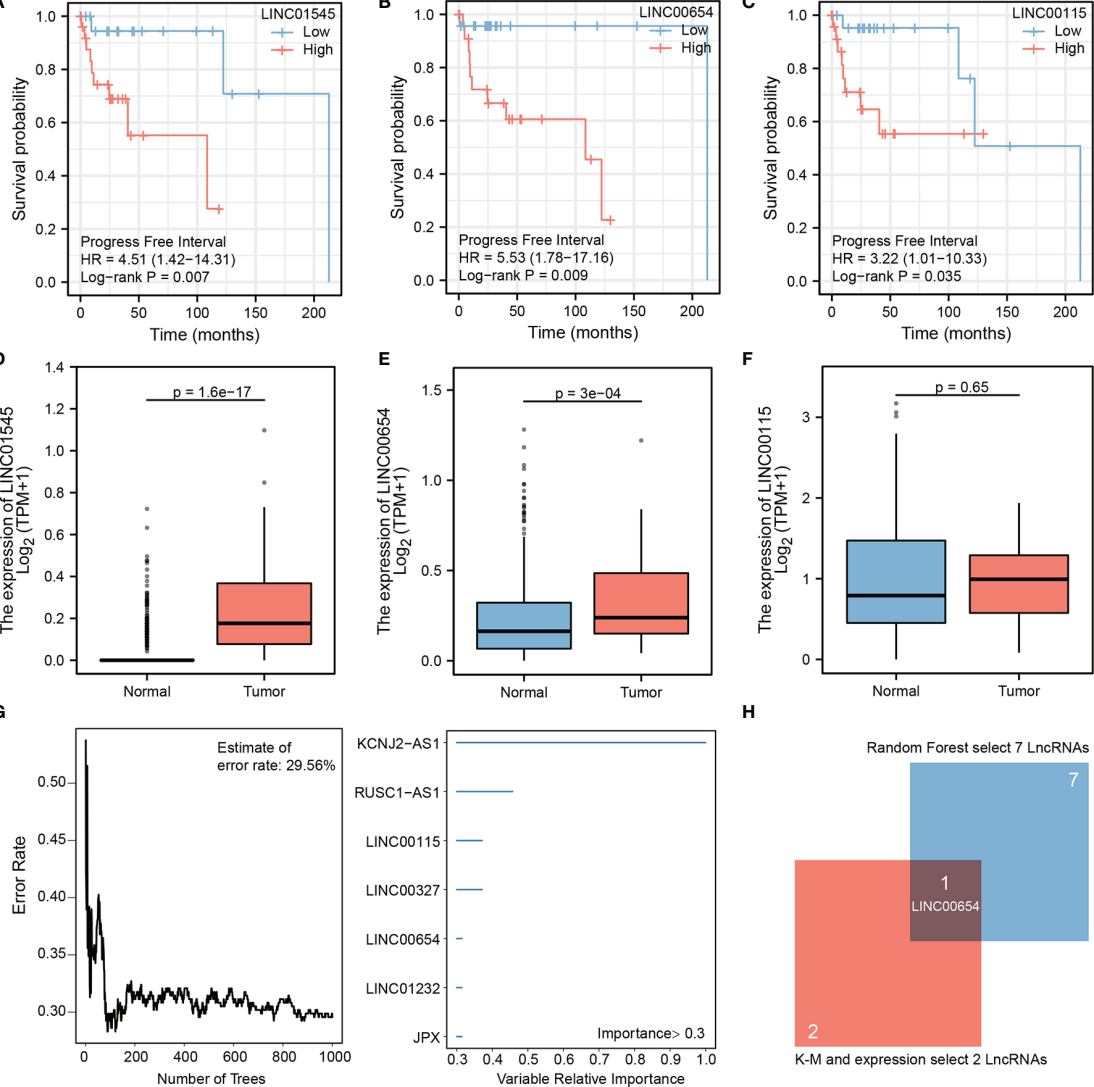
Hub lncRNA-LINC00654 identified using the public database. Batch Cox regression was performed to screen for independent risk lncRNAs, TOP three lncRNAs (lnc01545, lnc00654, and lnc00115) are selected **(A–C)**, expression validation of three lncRNAs in TCGA database, two candidate lncRNAs were identify (lnc01545 and lnc00654) **(D–F)**, random forest model was used to select important lncRNAs, the model error rate was 29.56%, seven lncRNAs are listed (KCNJ2-AS1, RUSC1-AS1, lnc00115, lnc00327, lnc00654, lnc01232, and JPX) **(G, H)** the Venn diagram shows that lnc00654 was identified as hub lncRNA.

Batch Cox regression analysis results showed that 23 significant lncRNAs correlated with the patients’ PFI (**Supplementary Table 1**). The top three significant lncRNAs were LINC01545 (HR=4.51, p=0.007), LINC00654 (HR=5.53, p=0.009), and LINC00115 (HR=3.22, p=0.035) (**Figures 1A–C**). In the TCGA and GTEx databases, LINC01545 and LINC00654 had higher expression in the tumor than the normal group, and the difference was statistically significant (p=1.6e-17 vs. p=3.e-04, respectively), while LINC00115 expression was similar between the two groups (p=0.65) (**Figures 1D–F**). Thus, LINC00115 was excluded from additional analyses.

### Identifying LINC00654 as a Hub LncRNA by Random Forest

Random forest was used to more accurately identify the hub lncRNA, and the trees were 1000. The results suggested that the model became robust when the estimated error rate was 29.56%. After limiting the importance threshold to 0.3, the following seven LncRNAs were selected: KCNJ2-AS1, RUSC1-AS1, LINC00115, LINC00327, LINC00654, LINC01232, and JPX (**Figure 1G**). These candidate genes along with LINC01545 and LINC00654 identified LINC00654 as the hub lncRNA that was most closely associated with patient outcomes (**Figure 1H**).

### Impact of Clinical Variables on LINC00654

**Figure 2:**
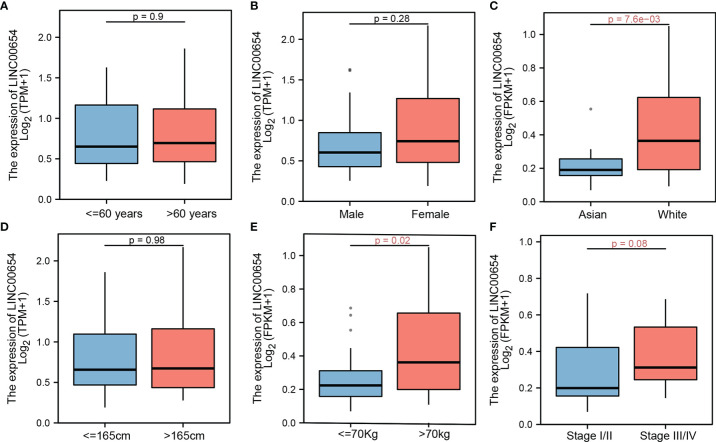
LINC00654 distribution by clinical factors. lnc00654 distribution in different clinical characteristics, in age and sex groups, no significance between groups, respectively (p=0.9 vs. p=0.28) **(A, B)**, lnc00654 expression significance between race groups (p=7.6e-03) **(C)** while no significance between the higher groups (p=0.98) **(D)**, during in weight and groups, lnc00654 expression high in patients with weight more than 70kg and with tumor in advanced stage (p=0.02 vs. p=0.08) **(E, F)**.

Clinical variables are important factors that affect patient outcomes. The correlation between LINC00654 distribution and common clinical characteristics was assessed. While LINC00654 distribution was similar within different age, sex, and height groups (p=0.9, p=0.28, and p=0.98, respectively), the distribution was significantly different within the race and weight subgroups (p=7.6e-03 vs. p=0.02, respectively) (**Figures 2A–E**). While the LINC00654 distribution showed no significance (p=0.08) between stages, expression was higher in stages III and IV than stages I and II (**Figure 2F**).

### Co-Expression Genes of LINC00654 and Enrichment Analysis

**Figure 3:**
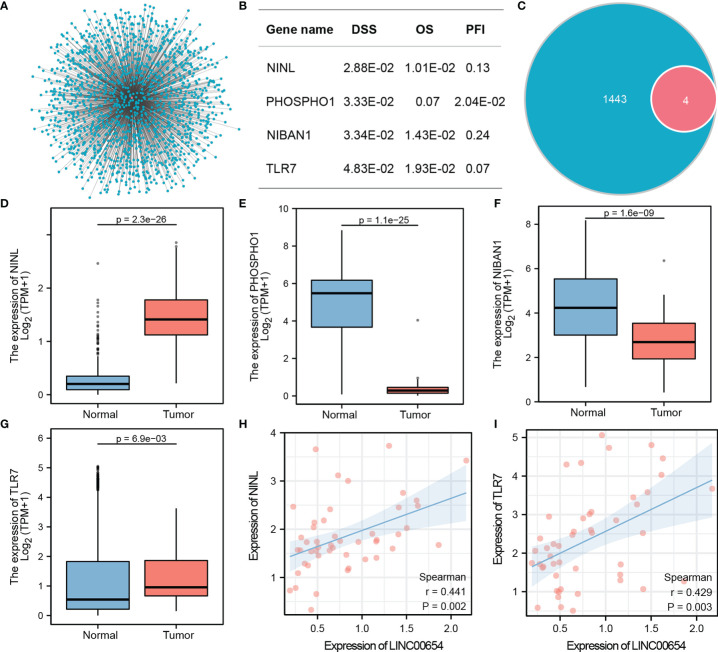
Identification of the Hub downstream target gene, NINL, of LINC00654. Co-expression genes of lnc00654, and batch cox regression analysis of all genes, four significant genes are list **(A, B)**. Venn figure shows the significant genes and target genes, expression validates genes between normal and tumor groups **(C–G)**. Correlation coefficients are used to screen the best candidates **(H, I)**.

Co-expression analysis using Spearman correlation was used to predict potential target genes, from which 1443 target mRNAs were selected (**Figure 3A** and **Supplementary Table 2**). The top 1000 target genes were used for GO and KEGG analysis, and the top five enrichment results are shown in **Tables 2**, **3**. GO analysis indicated that these genes appeared to be involved in regulating GTPase activity, focal adhesion, and Ras GTPase binding, while KEGG enrichment results suggested that they are involved in Notch and Hedgehog signaling as well as leukocyte transendothelial migration.

**Table 2:** Gene oncology enrichment of co-expression genes with LINC00654.

Items	GO	Description	Gene Ratio	p value
BP	GO:0010256	Endomembrane system organization	80/1351	9.34E-15
BP	GO:0043087	Regulation of GTPase activity	75/1351	1.57E-10
BP	GO:0048193	Golgi vesicle transport	60/1351	2.29E-09
BP	GO:0071902	Positive regulation of protein serine/threonine kinase activity	55/1351	7.44E-09
BP	GO:0043547	Positive regulation of GTPase activity	62/1351	1.49E-08
CC	GO:0010008	Endosome membrane	78/1411	5.64E-12
CC	GO:0005774	Vacuolar membrane	60/1411	1.08E-07
CC	GO:0030055	Cell-substrate junction	60/1411	1.08E-07
CC	GO:0005925	Focal adhesion	59/1411	1.36E-07
CC	GO:0045335	Phagocytic vesicle	28/1411	1.63E-07
MF	GO:0030695	GTPase regulator activity	57/1352	1.89E-10
MF	GO:0005096	GTPase activator activity	52/1352	6.62E-10
MF	GO:0017016	Ras GTPase binding	70/1352	1.03E-09
MF	GO:0031267	Small GTPase binding	71/1352	1.73E-09
MF	GO:0060589	Nucleoside-triphosphatase regulator activity	59/1352	3.23E-09

**Table 3:** KEGG pathway enrichment of co-expression genes with LINC00654.

Pathway	Items	Description	Gene Ratio	p value
KEGG	hsa05168	Herpes simplex virus 1 infection	77/637	3.71E-09
KEGG	hsa04810	Regulation of actin cytoskeleton	37/637	6.00E-06
KEGG	hsa04520	Adherens junction	18/637	6.42E-06
KEGG	hsa05132	Salmonella infection	38/637	5.40E-05
KEGG	hsa04330	Notch signaling pathway	13/637	1.78E-04
KEGG	hsa05131	Shigellosis	36/637	2.01E-04
KEGG	hsa04340	Hedgehog signaling pathway	12/637	3.89E-04
KEGG	hsa05130	Pathogenic Escherichia coli infection	29/637	7.44E-04
KEGG	hsa04919	Thyroid hormone signaling pathway	20/637	1.16E-03
KEGG	hsa04670	Leukocyte transendothelial migration	18/637	3.36E-03

### Identifying Prognosis-Related Downstream Targets of LINC00654

Batch Cox regression with 1443 candidate targets was used to evaluate the DSS. Four downstream genes were associated with DSS, NINL (HR=12.49, p=2.88E-02), PHOSPHO1 (HR=11.79, p=3.33E-02), NIBAN1 (HR=11.57, p=3.43E-02), and TLR7 (HR=9.84, p=4.83E-02). The prognosis value of these four genes was evaluated in the OS and PFI model, and the results confirmed that each of these genes was associated with two prognosis outcomes (**Figure 3B**). The four genes were selected for future analysis (**Figure 3C**).

### Selecting NINL as a Target Gene and Constructing the Regulation Axis

Two methods were used to select the hub downstream gene from four candidate targets. The expression of these genes between the tumor and normal groups was validated (**Figures 3D–G**). Results showed significant differences in the expression of these four genes between groups, however, NINL and TLR7 had the highest potential to become hub downstream targets, because their expression patterns matched LINC00654 while PHOSPHO1 and NIBAN1 had an opposite expression pattern. The correlation between either NINL or TLR7 and LINC00654 was also assessed. NINL correlated more strongly with LINC00654 than TLR7 (r=0.441, p=0.002, vs. r=0.429, p=0.003, respectively) (**Figures 3H, I**). Thus, NINL was selected as a hub downstream target of LINC00654. Based on these results, the LINC00654-NINL axis was also constructed.

### High Expression of the NINL Gene in DLBCL Patients and Tumor Cell Lines

**Figure 4:**
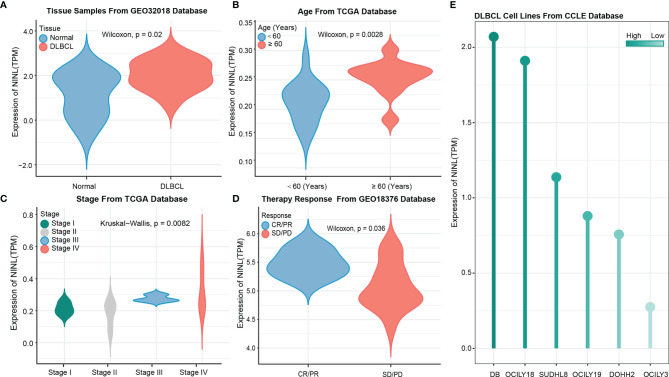
NINL expression validation in tissues and cell lines. NINL expression validation between normal and tumor groups from GEO dataset **(A)** and this gene expression validation in age and stage groups from TCGA dataset **(B, C)**, therapy response also validated in GEO dataset **(D)**. Expression in cell lines was performed in CCLE dataset **(E)**.

After constructing the regulation axis, NINL expression and clinical values were validated in independent datasets. GSE32018 dataset validation results showed that NINL expression differed significantly between tumor and normal tissues (**Figure 4A**), and subgroup analysis samples from TCGA datasets demonstrated that NINL expression was associated with age and stage. In the higher age group (>60 years of age) (p-value = 0.0028) and in advanced disease stages (p-value = 0.0028), patients exhibited increased expression of the NINL gene (**Figures 4B, C**). NINL gene expression was also verified in response to therapy, and a better response to therapy was associated with higher expression of this gene (p-value = 0.036) (**Figure 4D**). In addition, by analyzing the expression of cell lines from the CCLE database, NINL was expressed in six DLBCL cell lines, DB, OCILY18, SUDHL8, OCILY19, DOHH2, and OCILY3 (**Figure 4E**).

### Survival Analysis of NINL by Subgroup

**Figure 5:**
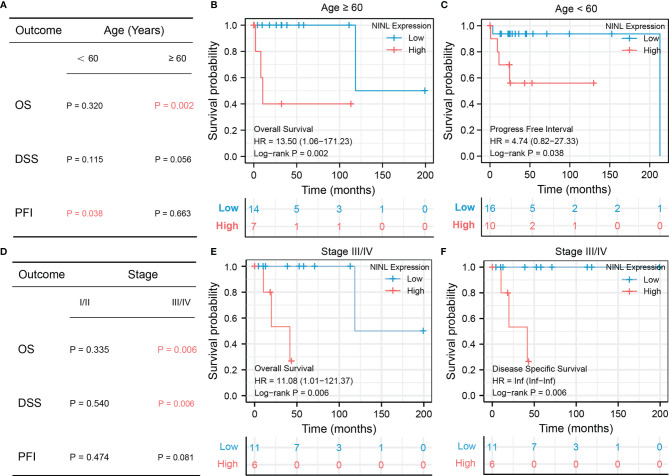
Subgroup survival analysis of NINL by age group and stage. Subgroup survival analysis of NINL by age groups, the significant outcome results are shown independent**(A–C)**, subgroup survival analysis of NINL by stage groups, the significant outcome results are shown independent **(D–F)**.

Because NINL expression differed significantly by age and stage, the prognostic value of the NINL gene was further compared between clinical subgroups. When stratifying by age, high expression of the NINL gene was found to be a risk factor for OS among patients ≥60 years of age (HR = 13.50, 95% CI (1.06–171.23), p-value = 0.002) and a risk factor for PFI among patients<60 years of age (HR = 4.74, 95% CI (0.82–27.33), p-value = 0.038) (**Figure 5A**). The survival curves are shown in **Figures 5B, C**. High expression of the NINL gene was shown to be a risk factor for OS and DSS among patients in the high stage group (p-value = 0.006 for both) (**Figure 5D**). The survival curve is shown in **Figures 5E, F**.

### Biological Functions of NINL and NINL-Related Pathways in DLBCL

**Figure 6:**
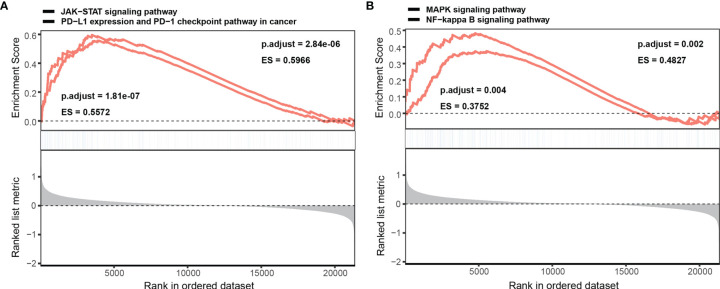
GSEA enrichment analysis of NINL. Single gene GSEA enrichment analysis of NINL, four significant pathways are listed **(A, B)**.

GSEA was performed to better understand the potential mechanism of NINL during DLBCL, and the top 10 significant genes involved in biological processes (BP), molecular function (MF), and cellular component (CC) enrichment analysis were assessed. For BPs, NINL was mainly enriched during neutrophil activation, neutrophil-mediated immunity, cell cycle arrest, and G2/M transition of the mitotic cell cycle. CC enrichment analysis showed that NINL was likely involved in focal adhesion and the formation of endosome membranes, microtubules, and the mitochondrial matrix. For MFs, NINL was mainly involved in small GTPase binding, Ras GTPase binding, and transcription factor binding to DNA (**Supplementary Table 3**). GSEA showed that NINL is significantly enriched during JAK-STAT signaling, PD-L1 expression and PD-1 checkpoint pathway in cancer, NF-κB signaling, and MAPK signaling (**Figures 6A, B**).

## Discussion

Long non-coding RNAs (lncRNAs) are a type of RNA that are defined as gene transcripts that are not translated into protein. Instead, they help to regulate their target protein-coding genes at multiple levels, including epigenetic regulation, transcriptional regulation, and post-transcriptional regulation, and are involved in the progression of different tumors (4, 5). Although some lncRNAs involved in DLBCL progression were identified using new sequencing technologies (9, 10, 22), the role of LINC00654 in DLBCL progression remains unknown. For the first time, LINC00654 was selected using a computational algorithm as the most important hub lncRNA associated with the PFI among patients with DLBCL, which is of significant diagnostic value. Xu et al. showed that LINC00654 is one of the lncRNAs with high diagnostic performance for CRC (11), and LINC00654 upregulation is also associated with poor breast cancer OS (8). Matboli et al. also showed that pentoxifylline can alleviate cardiac injury using the lncRNA-00654-miR-133a-SOX5 mRNA network (12).

Competing endogenous RNAs (ceRNAs) form a network between the function of protein-coding mRNAs and non-coding RNAs such as microRNA, lncRNA, pseudogenic RNA, and circular RNA (23). Because lncRNAs can regulate target mRNAs by post-transcription, it is of great significance to predict the interplay and explore the potential mechanisms of the pathological processes *via* the regulatory network. In 2020, Shi et al. (24) identified a novel ceRNA breast cancer network and used this to construct an effective signature to predict breast cancer outcomes. During the same period, the regulatory network of lncRNA and mRNA in skin cutaneous melanoma (SKCM) was characterized by analyzing expression profiling data, and potential treatment targets were identified (7). The current paper also identified a novel regulatory lncRNA-00654-NINL mRNA axis in DLBCL using bioinformatics and showed that this may provide a useful reference for exploring the mechanism of DLBCL progression.

The human NINL gene, located at chromosome 20p11, encodes the centrosomal protein, Nlp, a member of the γ-tubulin complex binding proteins (GTBPs) involved in mitosis. In mammals, Nlp expression is regulated by some cell cycle proteins and mitotic kinases such as PIK1, Aurora A, Cdc2, and Nek2 in a cell cycle-dependent manner (13, 14). The main function of Nlp is to promote the nucleation of microtubules, enabling centrosome maturation, spindle formation, and chromosome separation. The key step in formation of the centrosome and mitotic spindle is to remove the Nlp from the mature centrosome during the G2/M transition. In recent years, the stability of centrosome and mitotic events has been recognized as early factors in tumorigenesis, and Nlp is thought to play an important role as an oncogenic protein. Upregulated NINL mRNA and protein expression have been observed in several types of human tumors, such as head and neck squamous cell carcinoma (HNSCC), breast cancer, ovarian cancer, and lung cancer (16–21). Yu et al. found that the overexpressed NINL mRNA and protein expression in HNSCC tissue is strongly correlated with tumor grade (21). Another study showed that Nlp overexpression was associated with increased proliferation, invasion, and metastasis in MCF-7 cells, a breast cancer cell line, and reduced apoptosis in response to paclitaxel, likely because Nlp reduces microtubule aggregation and tubulin changes caused by this drug (17, 18). BRCA1, a breast cancer susceptibility gene related to cell cycle control, is also important for Nlp-mediated centrosome localization (16). While the rapid onset of radiation-induced lymphoma observed in NINL transgenic mice also confirms an oncogenic role for this gene, there is almost no information on how NINL expression impacts human tumor survival (20).

As shown in the TCGA cohort, high NINL expression predicted shorter OS and DSS than low expression. In addition, high expression of NINL was correlated with some clinical risk factors, such as age (> 60 years) and advanced stage (stage III or IV). These results indicate that NINL is an independent prognostic biomarker in DLBCL and could be a potential target for precision therapy. However, the 95% CIs for HR is too wide. This could be due to the sample size of the study, which may be affecting the generalizability of results. This conclusion needs to be validated in more clinical samples. On the other hand, the patients with upregulated NINL expression had a better response to therapy in another independent cohort (GSE18376), which differs from the poor prognostic results shown using TCGA data. Data on the response to therapy were unavailable in the TCGA-DLBCL database, however, one possible explanation is that most patients were given immunotherapy (rituximab) combined with chemotherapy, and the differences between the tumor immune microenvironments of the two cohorts may have caused bias.

More detail about the possible pathway and regulatory network of NINL in DLBCL needs to be elucidated. Nlp has been recognized as a centrosome protein and is likely to affect cell signaling and chromosomal stability in cancer cells. This mechanism is regulated by some cell cycle proteins and mitotic kinases (14, 19). The current study found that NINL is significantly enriched in cellular microtubules, and gene-set enrichment analysis (GSEA) revealed that NINL is significantly enriched in both the JAK-STAT and the NF-κB signaling pathways (25). DLBCL can be subdivided into three subtypes, germinal center B-cell (GCB), activated B-cell (ABC), and primary mediastinal B-cell lymphoma (PMBL) using gene-expression profiling (GEP) (26). In ABC-DLBCL, the NF-κB signaling pathway is constitutively activated and contains various transcription factors that can regulate immune and inflammatory responses, cell-cycle progression, and apoptosis (27). The IKK complex, a key regulator in the NF-κB signaling pathway, is shown to play a role in regulating the mitotic kinase, Aurora A. IKKα can phosphorylate Aurora A to regulate G2/M progression, and its stability can be deregulated by IKKβ, leading to the formation of abnormal spindles and chromosome missegregation (25, 28). Mutated MYD88 that is found in some ABC-DLBCL cases can activate MAPK and NF-κB signaling, which consequently activates JAK-STAT by upregulating IL-6 and IL-10 expression (29, 30). JAK-STAT activation induces some transcriptional targets, like cyclin D1 and p21, that are involved in cell cycle progression (31). As in GCB-DLBCL, the tumor suppressor, p53, and cell cycle checkpoint proteins, p21 and p27, are deregulated by activated BCL6, resulting in centrosome abnormality and cell-cycle progression (32, 33). Together these studies indicate that the cell-cycle regulated NINL gene may play a key role in the signaling pathways involved in DLBCL. Although NINL was enriched in different pathways through the LINC00654-NINL axis, this may be because it was only identified as one of the survival-related target genes of the hub lncRNA. More roles for LINC00654 in DLBCL remain to be defined.

This study has some limitations. First, the LINC00654-NINL axis in DLBCL should be verified using larger cohorts because the size of the TCGA database is limited. Second, the mechanisms of this axis during DLBCL require further exploration.

In conclusion, we identified the lncRNA-NINL mRNA regulatory axis as a novel regulation network in DLBCL. This axis may provide a useful reference for exploring the mechanism of DLBCL disease progression.

## Data Availability Statement

The original contributions presented in the study are included in the article/**Supplementary Material**. Further inquiries can be directed to the corresponding authors.

## Author Contributions

JQ and QP were involved in the conception and design of the study; YC, ZW, and CL analyzed the bioinformatic data; YC, CL, JZ, and NW drafted the manuscript; JY and YW reviewed and edited the manuscript. All authors have read and approved the final manuscript.

## Conflict of Interest

The authors declare that the research was conducted in the absence of any commercial or financial relationships that could be construed as a potential conflict of interest.

## Publisher’s Note

All claims expressed in this article are solely those of the authors and do not necessarily represent those of their affiliated organizations, or those of the publisher, the editors and the reviewers. Any product that may be evaluated in this article, or claim that may be made by its manufacturer, is not guaranteed or endorsed by the publisher.

## Supplementary Material

Supplementary Table 1 Batch Cox analysis of lncRNAs with prognosis outcome.

Supplementary Table 2 Co-expression target genes of LINC00654

Supplementary Table 3 TOP 10 items of NINL GO enrichment

## Abbreviations

lncRNA, long non-coding RNA; DLBCL, diffuse large B-cell lymphoma; GO, Gene Oncology; KEGG, Kyoto Encyclopedia of Genes and Genomes; GSEA, Gene Set Enrichment Analysis; NINL, Ninein like; OS, overall survival; DSS, disease-specific survival; NHL, non-Hodgkin lymphoma; RCHOP, rituximab, cyclophosphamide, doxorubicin, vincristine, and prednisone; Nlp, Ninein-like protein; TCGA, The Cancer Genome Atlas; GEO, Gene Expression Omnibus; CCLE, Cancer Cell Line Encyclopedia; PFI,Progression-free Interval; BP, biological processes; CC, Cellular components; MF, Molecular functions.
