# Supplementary material for: Identification of LINC00654-NINL Regulatory Axis in Diffuse Large B-Cell Lymphoma In Silico Analysis
Source: Front Oncol. 2022 May 26;12:883301. doi: 10.3389/fonc.2022.883301 (PMC9204339; doi:10.3389/fonc.2022.883301)
Supplement: Supplementary Table 1 — Batch Cox analysis of lncRNAs with prognosis outcome. [file Table_1.docx]

| **Supplement Table1**：Batch cox analysis LncRNAs with PFI | | | | | |
| --- | --- | --- | --- | --- | --- |
| gene_name | gene_id | gene_biotype | HR | CI | p.value |
| LINC01545 | ENSG00000204904 | lncRNA | 9.968 | 1.242-80.032 | 0.030498773 |
| LINC00654 | ENSG00000205181 | lncRNA | 9.62 | 1.227-75.436 | 0.031208041 |
| LINC00115 | ENSG00000225880 | lncRNA | 9.472 | 2.255-39.781 | 0.002134684 |
| KTN1-AS1 | ENSG00000186615 | lncRNA | 8.286 | 1.056-64.985 | 0.044197058 |
| PIK3CD-AS1 | ENSG00000179840 | lncRNA | 8.258 | 1.008-67.680 | 0.049185961 |
| KCNJ2-AS1 | ENSG00000267365 | lncRNA | 8.184 | 1.694-39.529 | 0.00888851 |
| RUSC1-AS1 | ENSG00000225855 | lncRNA | 7.992 | 1.597-39.984 | 0.011406423 |
| LINC01232 | ENSG00000280734 | lncRNA | 6.79 | 1.436-32.113 | 0.01568593 |
| SLFNL1-AS1 | ENSG00000281207 | lncRNA | 6.637 | 1.344-32.773 | 0.020173888 |
| LINC00327 | ENSG00000232977 | lncRNA | 6.157 | 1.320-28.727 | 0.020735032 |
| PAX8-AS1 | ENSG00000189223 | lncRNA | 5.853 | 1.507-22.726 | 0.010690923 |
| SNHG4 | ENSG00000281398 | lncRNA | 5.276 | 1.283-21.702 | 0.021177848 |
| CIRBP-AS1 | ENSG00000267493 | lncRNA | 5.135 | 1.317-20.024 | 0.018461464 |
| SPAG5-AS1 | ENSG00000227543 | lncRNA | 5.056 | 1.273-20.084 | 0.021305674 |
| COX10-AS1 | ENSG00000236088 | lncRNA | 4.967 | 1.033-23.881 | 0.045437068 |
| MIR600HG | ENSG00000236901 | lncRNA | 4.815 | 1.192-19.445 | 0.027313642 |
| SNHG1 | ENSG00000255717 | lncRNA | 4.321 | 1.082-17.263 | 0.038341955 |
| LINC00294 | ENSG00000280798 | lncRNA | 4.112 | 1.183-14.289 | 0.026104279 |
| BACE1-AS | ENSG00000278768 | lncRNA | 4.089 | 1.022-16.367 | 0.046569828 |
| PTGES2-AS1 | ENSG00000232850 | lncRNA | 4.079 | 1.081-15.393 | 0.038042969 |
| JPX | ENSG00000225470 | lncRNA | 4.055 | 1.123-14.647 | 0.032645436 |
| TSPEAR-AS2 | ENSG00000182912 | lncRNA | 3.94 | 1.149-13.513 | 0.029222896 |
| SNHG14 | ENSG00000224078 | lncRNA | 3.777 | 1.140-12.515 | 0.029688693 |
