# Supplementary material for: Identification of LINC00654-NINL Regulatory Axis in Diffuse Large B-Cell Lymphoma In Silico Analysis
Source: Front Oncol. 2022 May 26;12:883301. doi: 10.3389/fonc.2022.883301 (PMC9204339; doi:10.3389/fonc.2022.883301)
Supplement: Supplementary Table 3 — TOP 10 items of NINL GO enrichment [file Table_3.doc]

| **Supplement Table3 TOP 10 items of NINL GO enrichment** | | | | |
| --- | --- | --- | --- | --- |
| **Characteristics** | **ID** | **Description** | **GeneRatio** | **p.adjust** |
| BP^a^ | GO:0002283 | neutrophil activation involved in immune response | 462/14781 | 1.29E-22 |
| BP | GO:0007050 | cell cycle arrest | 221/14781 | 1.54E-09 |
| BP | GO:0000086 | G2/M transition of mitotic cell cycle | 229/14781 | 3.65E-09 |
| CC^b^ | GO:0005925 | focal adhesion | 390/15475 | 3.63E-25 |
| CC | GO:0010008 | endosome membrane | 446/15475 | 7.69E-19 |
| CC | GO:0005874 | microtubule | 377/15475 | 2.39E-11 |
| CC | GO:0005759 | mitochondrial matrix | 420/15475 | 1.39E-10 |
| MF^c^ | GO:0031267 | small GTPase binding | 419/14553 | 5.30E-15 |
| MF | GO:0017016 | Ras GTPase binding | 406/14553 | 1.04E-14 |
| MF | GO:0140297 | DNA-binding transcription factor binding | 333/14553 | 9.58E-13 |
| ^a^BP: biological processes, ^b^CC: cellular components,^c^MF: molecular functions | | | | |
